# Evaluation of hepatic fibrosis in HIV/HCV co-infected individuals in Yaoundé, Cameroon: usefulness of APRI score in resource-constrained settings

**DOI:** 10.1186/s12879-020-05477-7

**Published:** 2020-10-15

**Authors:** Rodolphe Dobseu, Aubin Nanfack, Mathurin Kowo, Georgia Ambada, Rachel Kamgaing, Collins Chenwi, Nadine Fainguem, Aude Ka’e, Eric Ngangoum, Samuel Sosso, Clergé Tchiegang, Alexis Ndjolo

**Affiliations:** 1 “Chantal Biya” International Reference Centre (CIRCB) for research on HIV/AIDS prevention and management, P.O. Box 3077, Yaoundé, Cameroon; 2 University of Ngaoundéré, Faculty of Sciences, Ngaoundéré, Cameroon; 3 University of Yaoundé I, Faculty of Medicine and Biomedical Sciences (FMSB), Yaoundé, Cameroon; 4 University Teaching Hospital (CHU), Yaoundé, Cameroon; 5 University of Rome Tor Vergata, Rome, Italy

**Keywords:** HIV/HCV co-infection, Liver fibrosis, APRI score, Thrombocytopenia, Resource constrained settings

## Abstract

**Background:**

HIV infection exacerbates the prognosis of HCV infection, with a faster progression of hepatitis. Hepatic fibrosis is the major disruption of the hepatic tissue architecture characterized by anarchic deposition and excess of the extracellular matrix. The objective of this study was to evaluate hepatic fibrosis in HIV/HCV co-infected individuals as compared to HCV mono-infected.

**Methods:**

A total of 97 participants (mean age 60.2 ± 14.3 years and 0.76 male/female sex ratio) was enrolled in a study conducted in Yaoundé, Cameroon from November 2018 to January 2019. Liver fibrosis was assessed by the APRI score (Aspartate Aminotransferase or AST/Platelet Ratio Index) which identifies the stage of fibrosis as classified by the Metavir system (F0 to F4). CD4 counts and plasmatic HIV viral load of HIV/HCV co-infected individuals were determined and the correlation between hepatic fibrosis and immuno-virological status established. Statistical analysis was done using Microsoft Excel 2016 and EpiInfo7 software.

**Results:**

A high proportion (63.6%) of HIV/HCV co-infected participants had an abnormal AST level: 73.6 ± 45.8 IU/L as compared to 58.5 ± 39.3 IU/L (59.3%) among HCV mono-infected participants. The frequency of thrombocytopenia was 63.6% with a mean platelet count of 137 ± 50 ×  10^3^ IU/L in HIV/HCV co-infected participants as compared to 176 ± 67 × 10^3^ IU/L in HCV mono-infected participants (38.4%). The progression of hepatic fibrosis in participants with clinically significant fibrosis: F2, F3 and F4 was higher among HIV/HCV co-infected and the mean APRI score was 1.7 ± 1.4 versus 1 ± 0.8 among HCV mono-infected (26.7%). All participants (100%) with detectable HIV viral load had clinically significant fibrosis compared to 33.4% in those with undetectable HIV viral load (*p* = 0.55). Only 42.9% participants with CD4 >  500 cells/μL had clinically significant fibrosis (*p* = 0.72) while 100% participants with CD4 <  200 cells/μL had clinically significant fibrosis (*p* = 0.58).

**Conclusions:**

A high level of AST combined with thrombocytopenia (APRI score > 1.5) is an indicator of hepatic fibrosis in HIV/HCV co-infected individuals. Because of its non-invasive and less costly nature, the APRI score can be a suitable biomarker to monitor hepatic fibrosis in HIV/HCV co-infected individuals in resource constrained settings.

## Background

Hepatitis C Virus (HCV) and Human Immunodeficiency Virus (HIV) infections are serious public health problems worldwide due to their high prevalence. In 2019, UNAIDS estimated that 37.9 million people worldwide were living with HIV (PLWHIV) [1]. Sub-Saharan Africa remains the most affected area with more than 70% of the world’s people affected [2]. About 3 to 4 million people are newly infected with HCV each year worldwide resulting in 71 million people with chronic infection, with nearly 400,000 deaths per year [3]. Although the available data is limited, it appears that HCV prevalence and mortality rates vary considerably across regions. Africa is the second most endemic area for HCV after the Middle East with an estimated prevalence of 3.2% [4]. HCV infection is widespread among HIV-infected populations because these two viruses share the same transmission routes (parenteral, sexual and mother-to-child). In a recent meta-analysis, approximately 2,278,400 individuals worldwide were co-infected with HIV/HCV, representing a prevalence of 6.1% of people infected with HIV [5].

Since the advent of Highly Active Antiretroviral Therapy (HAART) in 1996, HIV infection has become a chronic disease, with an increase in the life expectancy of HIV-infected individuals worldwide; from 1.2 million deaths in 2010 to 770,000 in 2019 representing a reduction of 33% since 2010 [1]. On the other hand, the quality of life and survival of people living with HIV are threatened by opportunistic infections among which the relatively frequent occurrence of chronic hepatitis C and the risks of hepatotoxicity under Antiretroviral Therapy (ART), leading to transaminase levels up to ten times higher than normal. Very often, the evolution of this chronic hepatitis C progresses to the stage of severe liver fibrosis, cirrhosis and Hepatocellular Carcinoma [6]. The prevention of the progression of chronic hepatitis C requires early diagnosis of hepatic fibrosis. Fibrosis, the consequence of the fibrogenesis process, is the major perturbation of the architecture of the hepatic tissue characterized by an uncontrolled and excessive deposition of the extracellular matrix. Although the mechanisms by which HIV and HCV interact to influence the progression of liver disease are not well understood, it has been reported that HIV infection increases HCV viremia by 2 to 8 times resulting in a decrease in spontaneous recovery from acute hepatitis. Secondly, HIV/HCV co-infection is responsible for increasing the risk of mother-to-child and sexual transmission of HIV and HCV (3 to 20% and 0 to 3%, respectively). Finally, HIV/HCV co-infection aggravates the histological course of HCV infection by accelerating the onset of hepatic fibrosis due to HCV [7].

In Cameroon, studies have estimated HCV prevalence between 1 and 13% [6] while HIV prevalence is 3.4% [8]. These data suggest that estimates of the prevalence of co-infection may vary depending on the study population and geographical area. Liver toxicity increases with multiple pharmacokinetic interactions in HIV/HCV co-infection, and low risk of HCV eradication continue to be a challenge for monitoring the rapid onset of liver fibrosis in HIV/HCV co-infected individuals as compared to HCV mono-infected. Indeed, mortality from liver disease remains even higher among HIV/HCV co-infected individuals than among HIV or HCV mono-infected individuals [9]. Since HCV screening and surveillance of people living with HIV (PLWHIV) is not systematic, there is little data in our context on the assessment of liver disease in HIV/HCV co-infection. However, the liver, in addition to being the target organ of HCV, hepatotoxic substances (drugs, alcohol), tumor and infectious manifestations, can also be the target organ of HIV [10]. This assessment should be systematic to prevent the progression of liver disease and initiate appropriate management. The objective of this study was to evaluate hepatic fibrosis in HIV/HCV co-infected participants as compared to HCV mono-infected using APRI score; In addition, we also evaluated the effect of HIV viremia and CD4 count on the occurrence of hepatic fibrosis in HIV/HCV co-infected participants.

## Methods

### Study design and setting

A cross-sectional and descriptive study to evaluate hepatic fibrosis in 97 participants followed at the Yaoundé University Teaching Hospital (CHUY) and the “Chantal Biya” International Reference Center (CIRCB) was conducted from November 2018 to January 2019. Participants included in this study were: (a) people with HCV chronic infection (presence of HCV-RNA 6 months after first diagnosis), (b) having given their consent, and (c) over 21 years of age. Socio-demographics were recorded using a questionnaire. Of note, all HIV infected participants were on antiretroviral therapy.

### Determination of AST level

The AST level was determined by spectrophotometry with the BT 3000 Plus equipment (Biotechnica Instruments, Rome-Italy) using the quantitative analytical method which consists in measuring the optical density of a given chemical substance generally in solution. The optical density of the samples is determined by a spectrophotometer previously calibrated over the absorption wavelength of the substance to be studied.

### Platelet count analysis

Platelet count was determined through full blood count testing with the Sysmex XN 1000 analyzer (Sysmex Corporation, Kobe-Japan) which allows the analysis of levels and the identification of human blood components by flow cytometry.

### Evaluation of hepatic fibrosis

The evaluation of hepatic fibrosis was determined using the APRI score (Aminotransferase divided by Platelet Ratio Index), which is a non-invasive technique that measures blood parameters (indirect markers of fibrosis such as AST and platelet count) [11]. Hepatic fibrosis was classified using the Metavir system on a scale of F0 to F4 (F0 = no fibrosis, F1 = minimal fibrosis, F2 = moderate fibrosis, F3 = severe fibrosis, F4 = cirrhosis) [12]. Fibrosis was considered clinically significant at the F2, F3 and F4 stages.

### CD4 analysis and quantification of HIV viral load

CD4 lymphocyte count was determined by flow cytometry using the CyFlow Counter (Sysmex Corporation, Kobe-Japan) which allows quantitative and qualitative analysis through identification and counting of CD4^+^ T lymphocytes according to the manufacturer’s instruction. The quantification of HIV viral load was determined by real-time PCR on the Abbott m2000rt platform (Abbott RealTime HIV-1, France) which is a quantification technique that allows the formation of amplified products to be monitored in real time, cycle by cycle, using fluorescent probes that are hybridized and activated simultaneously with amplification [13].

### Statistical analyses

Statistical analyses were done using Microsoft Excel 2016 and EpiInfo7 software. The correlation coefficient established the relationship between hepatic fibrosis and the immuno-virological status of the participants in this study. Multivariate analyses were used to assess the association between the dependent (APRI score) and independent variables (sex, age, viral load and CD4 cell count). A significant value was considered at *P* < 0.05.

## Results

### Characteristics of the study population

A total of 97 participants (11 HIV/HCV co-infected and 86 HCV mono-infected) were enrolled for this study. Most of the participants (57%) were female and the mean age of the study population was 60.2 years (IQR:21–85) as detailed in Table 1. The mean AST was 60.3 ± 40.1 IU/L [7 to 259] ranging from 73.6 ± 45.8 IU/L [26 to 179] in HIV/HCV co-infected participants to 58.5 ± 39.3 IU/L [7–259] in HCV mono-infected. The mean platelet was 172 ± 67 × 10^3^ IU/L [50 to 369 × 10^3^] ranging from 138 ± 50 × 10^3^ IU/L [57 to 227 × 10^3^ IU/L] in HIV/HCV co-infected participants to 176 ± 68 × 10^3^ IU/L [50 to 369 × 10^3^ IU/L] in HCV mono-infected. The incidence of thrombocytopenia in HIV/HCV co-infected participants was 63.6% compared to 38.4% in HCV mono-infected (see Table 1).

**Table 1:** Demographic and clinical characteristics of the study population

Variable	Study Population ***N*** = 97	HIV/HCV ***N*** = 11	HCV ***N*** = 86
**Gender n (%)**			
Female	55 (57)	07 (64)	48 (56)
Male	42 (43)	04 (36)	38 (44)
**Mean age in years**	60.2	58.9	60.4
**Mean AST (UI/L)**	60.3 (7–259)	73.6 (26–179)	58.5 (07–259)
**Mean platelet × 10**^**3**^ **(UI/L)**	172 (50–369)	138 (57–227)	177 (50–369)
**Mean APRI score**	1.1 (0.1–5.4)	1.7 (0.4–5.4)	1 (0.1–3.6)
Stage F0 n (%)	36 (37.1%)	03 (27.3%)	33 (38.4%)
Stage F1 n (%)	20 (20.6%)	01 (09.1%)	19 (22.1%)
Stage F2 n (%)	11 (11.3%)	00 (0.0%)	11 (12.8%)
Stage F3 n (%)	13 (13.4%)	04 (36.3%)	9 (10.5%)
Stage F4 n (%)	17 (17.5%)	03 (27.3%)	14 (16.2%)

### Evaluation of hepatic fibrosis outcomes

**Fig. 1:**
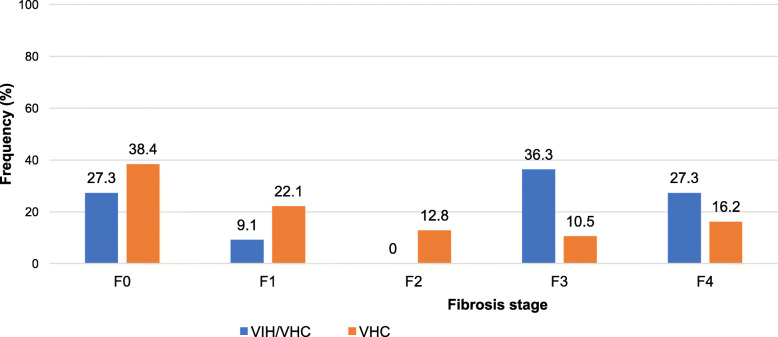
Distribution of the study population by stage of hepatic fibrosis. Classification Metavir system F0 to F4: F0 = no fibrosis; F1 = minimal fibrosis; F2 = moderate fibrosis; F3 = severe fibrosis; F4 = cirrhosis

**Fig. 2:**
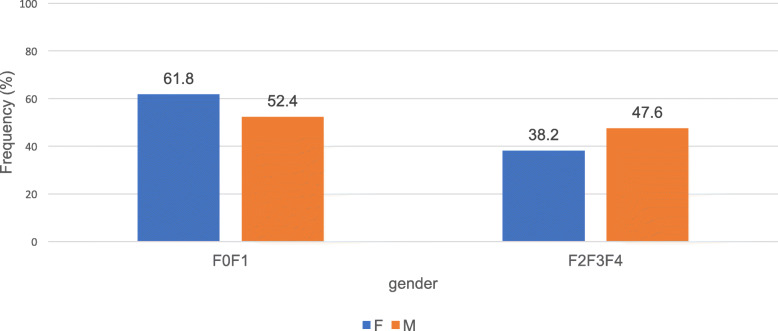
Distribution of hepatic fibrosis stage by gender. F0F1: Non-Clinically Significant Fibrosis; F2F3F4: Clinically Significant Fibrosis

The mean APRI score in this study was 1.1 ± 0.9 [0,1 à 5,4]. The mean APRI score for HIV/HCV co-infected participants was 1.7 ± 1.4 [0.4 to 5.4] and the frequency of participants with clinically significant fibrosis (F2, F3 and F4) per the Metavir classification was 63.6% (95% CI). The mean APRI score for HCV mono-infected participants was 1 ± 0.8 [0.1 to 3.6] and the frequency of participants with clinically significant fibrosis was 39.5% (95% CI). Figure 1 shows the distribution of the study population by stage of hepatic fibrosis and Fig. 2, the distribution of hepatic fibrosis stage by gender.

### Effect of HIV viremia on the progression of hepatic fibrosis

All HIV/HCV co-infected participants were on antiretroviral therapy and six (54.5%) had an undetectable HIV plasmatic viral load (< 40 copies/mL). All participants with detectable HIV viral load (≥ 40 copies/mL) had clinically significant fibrosis. Two (33.3%) participants with undetectable HIV plasmatic viral load had clinically significant fibrosis (Table 2).

**Table 2:** Distribution of APRI score by HIV viral load

HIV-CV	APRI Score Frequency (%)			
	F0	F1	F3	F4
Detectable	0 (0.00)	0 (0.00)	3 (60.00)	2 (40.00)
Undetectable	3 (50.00)	1 (16.67)	1 (16.67)	1 (16.67)
**TOTAL**	**3 (27.27)**	**1 (9.09)**	**4 (36.36)**	3 **(27.27)**

Legend: Detectable: Plasmatic HIV viral load > 40 copies/mL; Undetectable: Plasmatic HIV viral load < 40 copies/mL

### Effect of CD4 count on APRI score

The median CD4 count in this study was 536 cells/μL [106–875; IQR:169–709]. All participants with CD4 count < 200 cells/μL had clinically significant fibrosis. Three (42.86%) participants with CD4 >  500 cells/μL also had clinically significant fibrosis as shown in Table 3.

**Table 3:** Distribution of the population by CD4 count and APRI score

CD4 count	APRI Score Frequency n (%)			
	F0	F1	F3	F4
< 200	0 (0.00)	0 (0.00)	1 (33.33)	2 (66.67)
[200–350]	0 (0.00)	0 (0.00)	1 (100)	0 (0.00)
> 500	3 (42.86)	1 (14.29)	2 (28.57)	1 (14.29)
**TOTAL**	**3 (27.27)**	**1 (9.09)**	**4 (36.36)**	**3 (27.27)**

Legend: < 200: Severe immunodepression; [200–350]: Advanced immunodepression; > 500: Immunocompetent

### Predictors of the progression of hepatic fibrosis

Multivariate analysis considering HIV viral load, CD4 cell count, gender and age of participant’s, revealed that these factors were independently associated with the occurrence of hepatic fibrosis in the participants of this study. In participants over 60 years of age, the incidence of fibrosis stage is lower (45.3%) in those with clinically significant fibrosis than in those without. With respect to gender, the grade distribution of hepatic fibrosis was higher in men (47.6%) than in women (38.2%) with clinically significant fibrosis.

## Discussion

Subjects in the over-60 age group were the most infected with 63.6% of HIV/HCV co-infected subjects and 66.3% of HCV mono-infected subjects. These figures are different from those found in previous studies which found an average age of 48 [45–51] years old [14] and 38 [35–42] years old [15]. Given the silent evolution of HCV infection over several years, these results may be explained by the fact that in our context, screening for HCV infection is not systematic among high-risk such as HIV infected patients.

Serum aminotransferases (AST and ALT) are critical in the biological evaluation and surveillance of viral hepatitis C. Their increase for more than six-months period is a sign of a transition to chronicity, and 60 to 90% of chronic hepatitis with elevated transaminases progress to fibrotic liver disease unlike individuals with normal transaminases [10]. We found elevated mean AST level in HIV/HCV co-infected participants compared to HCV mono-infected (73.6 ± 45.8 IU/L versus 58.5 ± 39.3 IU/L). The increase in AST levels in our study population when compared to the normal AST value, which ranges between 5 to 40 IU/L in healthy individuals, could be explained by hepatic cytolysis since the liver is the main target organ for hepatic infections. Moreover, the increase in the rate of AST among HIV/HCV co-infected participants could also be linked to the increased replication of HCV-RNA activated by HIV infection; In fact, the interaction between HIV gp120 and CCR5/CXCR4 co-receptors on hepatocytes via TGF-β1 which is a key mediator in the process of liver fibrosis as it is one of the most profibrogenic cytokines [9]. Our results are consistent with a previous study that found that HIV/HCV co-infected individuals have a high level of AST ranging from 31 to 75 IU/L. [16].

The mean value of platelets in this study was 172 ± 67 × 10^3^ IU/L; almost similar to a previous study which found an average of 175 ± 79 × 10^3^ IU/L [16]. The incidence of thrombocytopenia in HIV/HCV co-infected participants is 63.6% as compared to 38.4% in HCV mono-infected. This incidence could be explained by the peripheral destruction of platelets due to HIV infection following the existence of antiplatelet autoantibodies specifically directed against certain antigenic determinants of the platelet membrane. The existence of these antiplatelet autoantibodies usually results from a cross-reactive humoral reactivity between viral and platelet components, in particular gp120 and gp IIb/IIIa [17, 18].

Using the Metavir system, 63.6% of our study participants co-infected with HIV/HCV had clinically significant fibrosis (F2, F3 and F4 stages) as compared to 39.5% mono-infected. These results are similar to previous studies that found F2, F3 and F4 stages in 59.8% of HIV/HCV co-infected individuals and 46.6% of mono-infected, respectively [19, 20]. This high rate of fibrosis is apparently link to the existence of a more active HCV infection supported by HIV-induced CD4^+^ T cell loss that deregulates T cell function leading to reduced anti-fibrotic activity of NK cells, resulting in accelerated progression of liver fibrosis in HIV/HCV co-infected participants [21, 22].

The correlation coefficient (r) between the liver fibrosis score and the age of patients in our study population (*r* = 0,06 with *p* = 0,04) shows statistically significant association between the two variables. This result is consistent with a previous study that showed a significant acceleration of hepatic fibrosis after the age of 50 years regardless of the age of infection [23]. Thus, age would be independently associated with the evolution of the liver fibrosis score.

Although the progression of significant fibrosis (F2, F3 and F4) is higher in men (47.6%) than in women (38.2%), we did not find a statistically significant association between these two variables (*P* = 0.11). On the other hand, a study has shown that women generally have a slower progression of significant fibrosis than men because estrogen has an inhibitory effect on fibrogenesis [23].

The decrease in CD4 count levels, due to HIV infection, leads to a reduction in the anti-fibrotic activity of NK cells, which leads to rapid progression of hepatic fibrosis in HIV/HCV co-infected individuals [9]. The median CD4 count in this study was 536 cells/μL [IQR:169–709] which is similar to a previous study that found a median CD4 count of 584 cells/μL [IQR: 396–775] in HIV/HCV co-infection [14]. All study participants (100%) with CD4 <  200 cells/μL had clinically significant fibrosis; therefore, supporting the fact that the decrease in CD4 count levels may activate viral replication C, resulting in increased destruction of liver cells leading to evolution of the fibrosis stage as shown in other studies [9]. The association between the fibrosis score and CD4 count was not statistically significant (*P* = 0.58).

The evolution of HIV infection leads to a strong presence of lipo-saccharide proteins which activate hepatic stellar cells inducing the progression of hepatic fibrosis [10]. Therefore, high viral load can lead to clinically significant fibrosis as exemplified in this study where all participants with detectable HIV viral load had clinically significant fibrosis versus 33.3% of participants with undetectable viral load; though we did not observe a statistically significant association between liver fibrosis score and HIV viral replication (*P* = 0,55). This result is similar to an earlier study that found no association between HIV viral load and APRI score in individuals co-infected with HIV/HCV [14].

### Limitations

Histologic study (liver biopsy) remains the gold standard method to evaluate liver fibrosis stage, unfortunately we were unable to perform it due to lack of funds. We used APRI score to evaluate liver fibrosis in HCV/HIV co-infected patients while it has been widely validated only in HCV mono-infected patients. However, all co-infected participants were on highly active antiretroviral therapy (HAART) which is known to control HIV infection and significantly reduces the impact of HIV in co-infections. It would be interesting in future studies to performed liver biopsy for HCV mono-infected and co-infected HIV/HCV patients to better set APRI index in both cases.

## Conclusion

The occurrence of higher than normal AST elevation and thrombocytopenia leads to an increased APRI score, a useful tool to stratify the risk of fibrosis progression in HCV infected individuals. Because of its non-invasive and less costly nature, the APRI score can be used as a biological marker to monitor HIV/HCV co-infected individuals in resource limited settings.

## Data Availability

The datasets used and/or analysed during the current study are available from the corresponding author on reasonable request.

## Abbreviations

AIDS: Acquired Immune Deficiency Syndrome
APRI: AST/Platelet Ratio Index
CHU: University Teaching Hospital
CIRCB: “Chantal Biya” International Reference Centre
HAART: Highly Active Antiretroviral Therapy
HCV: Hepatitis C Virus
HIV: Human Immunodeficiency Virus
PLWHIV: People Living With HIV
UNAIDS: United Nations Acquired Immune Deficiency Syndrome
